# Supplementary material for: Colistin kills bacteria by targeting lipopolysaccharide in the cytoplasmic membrane
Source: eLife. 2021 Apr 6;10:e65836. doi: 10.7554/eLife.65836 (PMC8096433; doi:10.7554/eLife.65836)
Supplement: Supplementary file 1. [file elife-65836-supp1.docx]

**Supplementary file**

**Colistin kills bacteria by targeting lipopolysaccharide in the cytoplasmic membrane**

Akshay Sabnis, Katheryn L. H. Hagart, Anna Klöckner, Michele Becce, Lindsay E. Evans, R. Christopher D. Furniss, Despoina A. I. Mavridou, Ronan Murphy, Molly M. Stevens, Jane C. Davies, Gerald J. Larrouy-Maumus, Thomas B. Clarke and Andrew M. Edwards

**This file includes:**

Supplementary Table 1


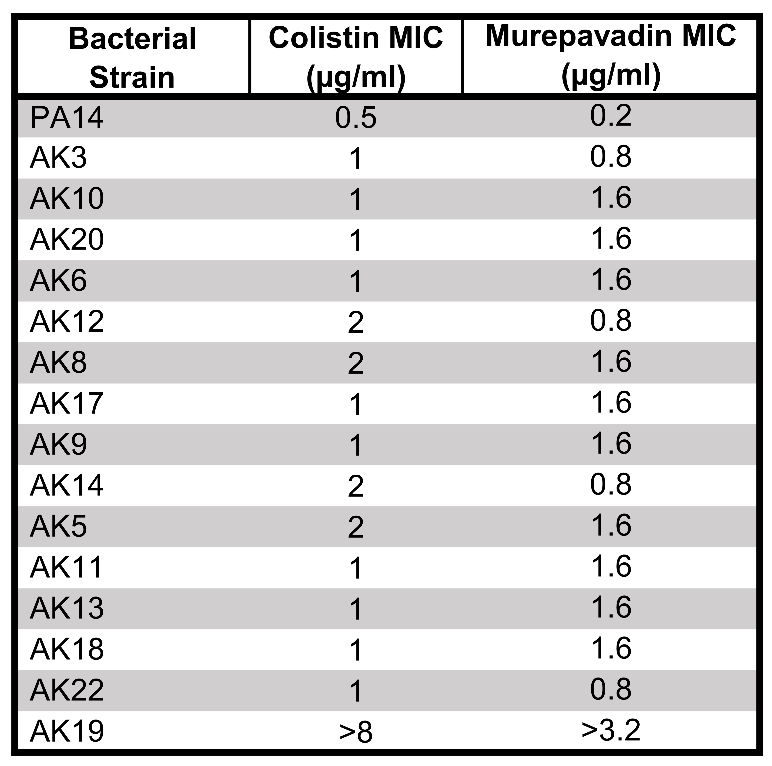


**Supplementary Table 1. Minimum inhibitory concentrations (MICs) of colistin and murepavadin against the *P. aeruginosa* strains used in this study.** MICs were determined in cation-adjusted Mueller-Hinton Broth (CA-MHB) using the broth microdilution method, as previously described [66]. The MIC was defined as the lowest antibiotic concentration at which there was no visible growth of bacteria after 18 hours incubation. Data are presented as the median of at least 3 independent biological replicates.
